# Perceptions on the Reasons Influencing the Choice and Abandonment of Exclusive Breastfeeding in Muslim and Christian Women: A Phenomenological Study

**DOI:** 10.1111/nhs.70195

**Published:** 2025-07-22

**Authors:** Isabel del Mar Moreno‐Ávila, Jose Manuel Martínez‐Linares, Sandra García‐Pintor, Susana María Rubia‐Ortega, Judith Ortiz‐Paneque, Shausan El Messaoudi‐Ouardani, Jonathan Cortés‐Martín

**Affiliations:** ^1^ Centro de Salud Zona Centro Gerencia de Atención Primaria, Instituto de Gestión Sanitaria Melilla Spain; ^2^ Departamento de Enfermería Facultad de Ciencias de la Salud, Universidad de Granada Granada Spain; ^3^ Unidad de Maternidad Hospital Universitario Poniente, Servicio Andaluz de Salud El Ejido Almería Spain; ^4^ Centro de Salud San Isidro Gerencia de Atención Primaria, Servicio Canario de la Salud Santa Cruz de Tenerife Spain; ^5^ Hospital Comarcal de Melilla Instituto de Gestión Sanitaria Melilla Spain; ^6^ Departamento de Enfermería, Facultad de Ciencias de la Salud Universidad de Granada Melilla Spain

**Keywords:** breast feeding, health personnel, life, phenomenology, qualitative research, religion

## Abstract

Exclusive breastfeeding is the best form of nutrition due to its benefits for the infant, the mother, society, and the environment. Despite its promotion, there are still barriers to making it a reality. To understand the reasons influencing the choice and abandonment of exclusive breastfeeding in women of Christian and Muslim religious beliefs. Descriptive phenomenological qualitative study. Two groups of women, one Muslim and one Christian. Four focus groups and in‐depth interviews were conducted, recorded, transcribed, and analyzed using ATLAS.ti 9.0. An inductive analysis was carried out according to Moustakas' model. All participants signed the corresponding informed consent form. This study adhered to the consolidated criteria for reporting qualitative research. Four themes were identified: (1) sociodemographic characteristics related to breastfeeding, (2) religious belief and cultural influence on breastfeeding type, (3) type of delivery and breastfeeding type, and (4) knowledge about breastfeeding and support received. The variety of existing reasons may vary according to the mothers' religious beliefs, requiring nursing interventions to be adapted to the characteristics of the women and families they serve.

**Summary**

- What does this paper add?
  ○ Religious beliefs are factors that influence when a woman becomes pregnant and modify her perception of breastfeeding.
  ○ The results provide valuable guidance for future interventions to strengthen breastfeeding care and attention for women with different religious ideologies.
  ○ The findings of this study may help improve the care provided to women and families in some aspects of exclusive breastfeeding.

## 1 Introduction

If all infants aged 0–6 months were optimally breastfed, more than 820 000 lives could be saved each year (World Health Organization [WHO] 2023a, 2023b). Globally, only 44% of infants are exclusively breastfed during the first 6 months of life (United Nations International Children's Emergency Fund [UNICEF] 2022). In Spain, 90.7% of women choose to start breastfeeding at birth, but only 35.2% maintain it at 6 months (Martín‐Ramos et al. 2024). Infants exclusively breastfed for at least the first 6 months have a 60% lower risk of sudden infant death syndrome, a 13% lower risk of overweight and obesity, and a 35% lower risk of type 2 diabetes compared to those who are not (Pan American Health Organization [PAHO] 2023).

International organizations and professional societies recognize the importance of the role of nursing and midwifery staff in promoting and improving exclusive breastfeeding rates, as well as in helping women achieve it (Global Breastfeeding Collective 2021; La Leche League International 2020; International Confederation of Midwives 2023). Hence, the importance of understanding mothers' experiences regarding the reasons for choosing and abandoning exclusive breastfeeding.

Exclusive breastfeeding is the practice of feeding the infant only with breast milk during the first 6 months of life (without giving any other food or even water) (World Health Organization 2014). Exclusively breastfed infants have a 73% lower risk of overweight and obesity (Horta et al. 2023), a 90.3% improvement in motor development (Hernández‐Luengo et al. 2022), an 82% lower risk of malnutrition (Mardani et al. 2022), present an 82% lower risk of malnutrition (Mardani et al. 2022), or a 56% lower risk of developing respiratory disease (Dib et al. 2024). Similarly, exclusive breastfeeding for women results in a 30% and 13% lower risk of developing diabetes mellitus and hypertension respectively (Rameez et al. 2019), increases the reduction of ovarian cancer risk as its duration increases (Eoh et al. 2021) regardless of parity (Babic et al. 2020), and is a protective factor against cardiovascular risk in women who develop gestational diabetes (Pathirana et al. 2022), as well as against cardiovascular disease in general (Tschiderer et al. 2022). However, there is no evidence of the protective effect of breastfeeding on the development of mental health problems in either mothers or their infants (Bugaeva et al. 2023).

Despite existing evidence, barriers persist that hinder the promotion of exclusive breastfeeding by healthcare professionals. These barriers include: lack of human, material, and economic resources; lack of preparation of mothers on exclusive breastfeeding before childbirth (Ramos‐Morcillo et al. 2020), lack of properly trained healthcare staff to carry out this task (Wu et al. 2024; Baker et al. 2021), cultural practices that maintain the idea that exclusive breastfeeding is insufficient to satisfy the baby's hunger and thirst (Pemo et al. 2020), or the lack of workplace support for women to continue practicing this type of breastfeeding (Hookway and Brown 2023).

The WHO (2018) published the 10 Steps to Successful Breastfeeding, which includes a package of policies and procedures that maternal and child health care facilities should implement to support exclusive breastfeeding. The interventions that have shown the greatest effectiveness in promoting exclusive breastfeeding are the implementation of the Baby‐Friendly Hospital Initiative, skin‐to‐skin contact, kangaroo mother care, cup feeding, and continuity of care and support in community and family settings through home visits (Tomori et al. 2022). A combination of strategies is necessary (Chipojola et al. 2022), especially in the case of younger mothers (Buckland et al. 2020). All of this can increase the rates of exclusive breastfeeding during the first 6 months of life (Dib et al. 2024).

Programs for promoting exclusive breastfeeding conducted by midwives have proven useful, but they must incorporate counseling skills regarding the reasons influencing women in the choice and abandonment of exclusive breastfeeding (Wang et al. 2023). For this purpose, having the qualification as an International Board Certified Lactation Consultant is beneficial (Walsh et al. 2023). However, barriers and issues still exist that hinder the increase in exclusive breastfeeding rates, resulting in economic losses of 302 billion dollars annually, as well as losses in benefits for the baby, the mother, society, and the environment (Rollins et al. 2016).

People can conceive and live their religious beliefs as part of their being holistically, and not taking this into account in clinical practice can be considered a lack of interest, becoming a barrier to communication and trust‐building (Aggarwal et al. 2022). It has been found that exclusive breastfeeding rates at birth differ according to the mother's religious beliefs, being higher among Muslim women compared to Christian women (Sharma et al. 2023). Therefore, it is important to understand the perceptions of women of different religious beliefs about the reasons influencing the choice and abandonment of exclusive breastfeeding.

The aim of this study was to understand the reasons influencing the choice and abandonment of exclusive breastfeeding among women of Christian and Muslim religious beliefs.

## 2 Methods

### 2.1 Design

A qualitative phenomenological descriptive study was conducted according to Husserl's approach. Phenomenological methodology is suitable for describing people's experiences regarding a phenomenon (Creswell 2005), and the Husserlian approach is indicated for nursing research (Frechette et al. 2020; Thomas 2021). In Health Sciences, phenomenology allows understanding the relationship between lived experience and health (Holloway and Wheeler 2010). In this study, it allowed exploring the meaning of the reasons influencing the choice and abandonment of exclusive breastfeeding in a sample of women in their life context. The phenomenon studied was the lived experience of mothers of two different religious beliefs during the breastfeeding period, a time when spirituality is present. The descriptive phenomenological approach made it possible to describe participants' subjective experiences and uncover determining aspects of this phenomenon. This was possible because the research team excluded their preconceived experiences through eidetic reduction and described the phenomenon from a free and impartial position in a second phenomenological reduction (Husserl 1992). This study lasted 2 years, and the Consolidated criteria for reporting qualitative research (COREQ) (Tong et al. 2007) were followed to prepare the report.

### 2.2 Theoretical Framework

The development of cultural competence is a tool for healthcare professionals to improve care for people from different cultures (Ross et al. 2022). This also applies to the promotion of exclusive breastfeeding (Hassan et al. 2020), becoming a demand from women in different societies who must decide what type of breastfeeding they want for their children (Rehayem et al. 2020). In this regard, Spector (1979) is the reference model that nursing staff must apply in multicultural societies, as it considers health‐related practices and beliefs that influence the experiences of people from different cultural backgrounds (Spector 2002). In breastfeeding, these cultural practices and beliefs can also be reflected (Pemo et al. 2020). Therefore, the promotion of breastfeeding must consider religious and cultural factors that will affect not only its initiation but also its maintenance (Mirafzali et al. 2022). In this respect, Gyamfi et al. (2023) attribute a significant role to religious beliefs and sociocultural practices in maintaining breastfeeding.

### 2.3 Study Setting and Recruitment

The research was conducted in Melilla (Spain), a city in North Africa where people of Christian (46% of the population), Muslim (52%), and Jewish beliefs coexist (Georgetown University 2020). The study was conducted between November 2022 and November 2023. Two groups of participants were included: one of Muslim religious beliefs and one of Christian. All of them were postpartum women. They were recruited in the Women's Health Unit, where pregnancy and postpartum controls are performed for women (both of Spanish origin and other nationalities) affiliated with the Spanish social security system, holding a residence card, or entitled to pregnancy, childbirth, and postpartum follow‐up according to Organic Law 4/2000 on the rights and freedoms of foreigners in Spain and their social integration. In the city of Melilla, there is only one hospital and four health centers serving the entire population. All of them belong to the public health system, which is used by the population regardless of their religious beliefs (Institute of Health Management 2023). Similarly, professionals of all categories with different religious beliefs work together in the public health system without any problems with coexistence. The study sample consisted of 17 Muslim women and 17 Christian women (Table 1).

**TABLE 1:** Sociodemographic characteristics of the participants (*n* = 45).

Code	Age at first birth	Parity (number)	Religious beliefs	Practicing	Type of breastfeeding	Antenatal classes
1A	32	1	Christian	Yes	EBF	Yes
2Y	23	1	Muslim	Yes	EBF	Yes
3L	32	1	Christian	Yes	MBF	Yes
4N	30	1	Muslim	Yes	EBF	No
5M	36	1	Christian	No	MBF	Yes
6Y	29	2	Muslim	Yes	EBF	No
7M	34	2	Christian	No	EBF	Yes
8L	21	4	Muslim	Yes	EBF	No
9A	39	1	Christian	Yes	EBF	Yes
10D	29	2	Muslim	Yes	EBF	No
11A	31	1	Christian	No	EBF	Yes
12D	22	3	Muslim	Yes	EBF	No
13M	35	1	Christian	Yes	MBF	Yes
14K	23	2	Muslim	No	MBF	Yes
15L	25	2	Christian	No	EBF	Yes
16A	22	5	Muslim	Yes	EBF	No
17M	31	1	Christian	Yes	EBF	Yes
18L	27	2	Muslim	No	MBF	No
19M	32	1	Christian	No	ABF	No
20N	23	3	Muslim	Yes	EBF	Yes
21A	34	1	Christian	Yes	MBF	No
22H	24	2	Muslim	Yes	EBF	No
23E	29	2	Christian	No	EBF	Yes
24H	33	3	Muslim	Yes	EBF	No
25C	36	1	Christian	No	MBF	Yes
26Y	28	3	Muslim	Yes	EBF	No
27C	31	1	Christian	No	MBF	Yes
28S	24	5	Muslim	Yes	EBF	No
29C	32	1	Christian	Yes	EBF	Yes
30H	22	3	Muslim	Yes	MBF	No
31C	28	2	Christian	No	EBF	Yes
32M	32	2	Muslim	Yes	MBF	No
33T	29	1	Christian	No	ABF	No
34S	27	2	Muslim	Yes	EBF	Yes

Abbreviations: ABF, artificial breastfeeding; EBF, exclusive breastfeeding; MBF, mixed breastfeeding.

### 2.4 Inclusion and/or Exclusion Criteria

Participants were recruited through intentional sampling during the third pregnancy visit (24–25 weeks of gestation) to avoid late abortions, and according to the inclusion criteria: aged between 18 and 45 years, residing in Melilla, and having received pregnancy, childbirth, and postpartum follow‐up within the public health system. Women with maternal–infant health problems that influenced breastfeeding or those who refused to participate (due to their reluctance to express their opinions and experiences about their religious beliefs) were not included. The only relationship with the participants was the professional one generated during pregnancy follow‐up. Women only participated in the focus groups that were conducted or were interviewed, but never in both data collection methods.

### 2.5 Data Collection

The data were collected in the city of Melilla as follows: First, two focus groups were conducted with a total of 10 women in each group (5 of Muslim belief and 5 Christian). These women were recruited through purposive sampling at the midwife's prenatal clinic (the midwife had a master's degree). This is a known place for the women, and at that time, they were informed about the study that was to be conducted. The focus groups were organized when the women attended their postpartum visit and were held at least 4 months after childbirth. Each focus group lasted approximately 1 h and served as an initial approach to the study topic and their experiences with breastfeeding. The sessions began with the open question, “Tell me in as much detail as possible everything that you think has been important and is related to the breastfeeding of your recent daughter/son.” Two researchers were present in the focus groups, with one acting as an observer, recording nonverbal communication elements in a field notebook that were later incorporated into the data analysis.

The collected data were used to conduct in‐depth interviews, as a second data collection method, 1 month later. These were conducted in the same location with women recruited in the same way, who had not participated in the focus groups. The interview script was developed with aspects related to breastfeeding that were highlighted in the focus groups. The interviews were conducted and started with the same open question used in the focus groups. Absolute freedom was allowed in the responses to create a calm and peaceful atmosphere. The interviews were audio‐recorded and transcribed verbatim. The transcripts were sent to the participants to verify their accuracy. Data saturation was achieved with 14 interviews conducted (Morse 2015). Table 2 shows the interview protocol.

**TABLE 2:** Interview protocol.

Interview stage	Topic	Example question
Introduction	Reasons	Belief that their perspective provides knowledge that should be known globally.
	Intention	Belief that their perspective provides knowledge that should be known globally. Conduct research with the aim of showing a real situation.
Beginning	Initial question	Tell me in as much detail as possible everything that you think has been important and is related to the breastfeeding of your recent daughter/son.
Development	Clarification prompts	Do you think there are factors that may have influenced the choice of type of breastfeeding? How do you think breastfeeding is influenced by religious belief in a context like the city of Melilla?
Conclusion	Final question	Is there anything else you would like to tell me?
	Appreciation	Thank you very much for your participation. Your contributions are of great value. I would like to remind you that you can call or email me if you have any questions or need further clarification.
	Offer	I would like to remind you that you can call or email me if you have any questions or need further clarification.

### 2.6 Data Analysis

The textual transcripts were read by two researchers, who listened to them again to better understand the experiences described by the participants and included non‐verbal communication elements. An inductive analysis was conducted following Moustakas' (1994) model, which is a modification of the Stevick‐Colaizzi‐Keen method commonly used in descriptive phenomenological studies. First, a complete reading of the transcripts was performed. Then, a second reading was conducted to extract units of meaning and create categories by grouping them according to the relationships established between them, thus coding the information. In the next step, the categories were grouped into themes. For each theme identified in each interview, a textual or general description of the experiences described by the participants was made. Next, a structural description of each theme was developed for each interview, followed by a textual‐structural description of each interview. Finally, all were integrated, generating the identified themes.

The data analysis was carried out individually by two researchers, who then compared their results. The findings were returned to the participants to identify their contributions and allow them to make inputs to the researchers' interpretation. ATLAS.ti 9 software for Windows (Scientific Software Development GmbH, Berlin, Germany) was used.

### 2.7 Ethical Considerations

The ethical principles of the Declaration of Helsinki were applied, and the confidentiality of personal data was respected according to Organic Law 7/2021, of May 26, on data protection. Authorization was obtained from the coordinator of the Women's Care Unit of the Zona Centro Health Centre (Melilla Health Area, Spain) before data collection began (November 4, 2019). The Melilla Health Area does not have a research ethics committee. Before participating, all women signed the corresponding informed consent form. The confidentiality of personal data was ensured by assigning alphanumeric codes to each participant in both the interviews and focus groups, in accordance with Organic Law 3/2018, of December 5, on the protection of personal data and digital rights.

### 2.8 Rigor and Reflexivity

The scientific rigor of the study was provided by the quality criteria of Lincoln and Guba (1985). Credibility was achieved through data saturation in both the focus groups and the interviews; data collection triangulation through interviews and focus groups, researcher triangulation for data analysis, and returning the transcripts to the participants. Transferability was ensured with the large amount of detail provided about the context and the participants' sociodemographic characteristics. Dependability is demonstrated in the detailed description of the methodology and the consistency between the design type, data collection, and type of analysis conducted. Confirmability is manifested through the feedback obtained from the participants (both in the transcripts and the results) and the incorporation of direct quotes from their accounts in the Results section.

## 3 Findings

**FIGURE 1:**
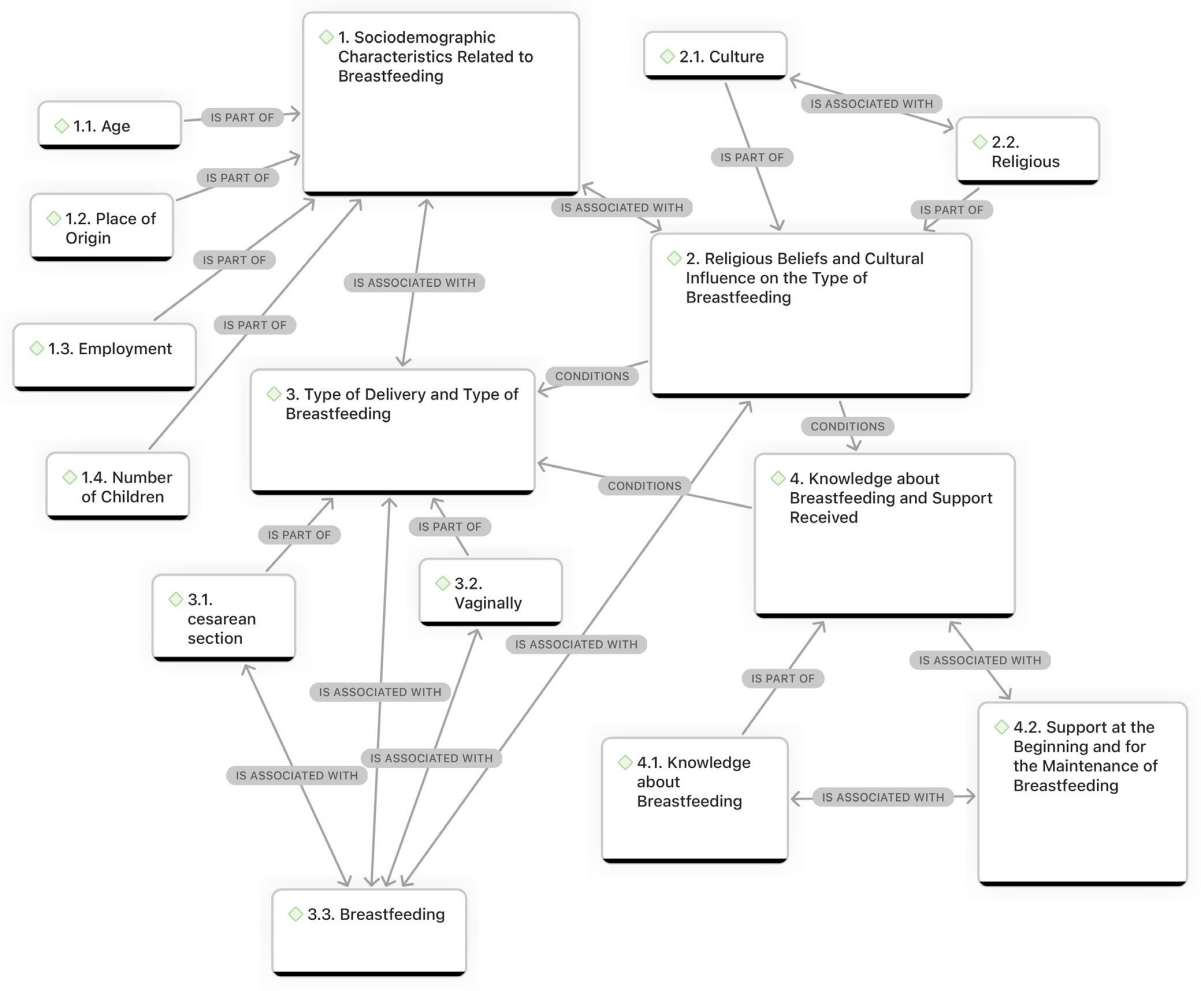
Conceptual map. *Source:* Atlas.TI version 9.

The data analysis identified four themes that addressed the study's objective: (1) Sociodemographic characteristics related to breastfeeding, (2) Religious beliefs and cultural influence on the type of breastfeeding, (3) Type of delivery and type of breastfeeding, and (4) Knowledge about breastfeeding and support received (Figure 1).

### 3.1 Theme 1: Sociodemographic Characteristics Related to Breastfeeding

#### 3.1.1 Age

Both Christian and Muslim women who became mothers at younger ages showed greater concern about all issues associated with pregnancy and a greater interest in breastfeeding. However, those women who had their first childbirth before the age of 20 were not clear about what type of breastfeeding they wanted to choose and leaned more toward mixed or artificial breastfeeding.

My first childbirth was at 29 years old. Both my partner and I have been involved in the pregnancy, reading and attending maternal education classes. Now with the little one, we are a bit overwhelmed, but it's normal. I am exclusively breastfeeding and very happy. We read a lot about it and knew that if we wanted to and everything went well, it could be achieved. (25C)

#### 3.1.2 Place of Origin

Women who were not from Melilla described the environment of this city as very closed, “very small town.” They work in Melilla, but whenever they can, they return to their place of origin. They make a comparison between both places and perceive that the family, social, and cultural environment they live in influences both how they experience pregnancy and the type of breastfeeding they decide to practice.

It's a small‐town city, and everyone knows everything about everyone. Although my island is also small, perhaps the way of life is more bohemian or liberal, and that's what I am used to and really need now. My daughter was born 12 days ago, and next weekend we are leaving. (3L)

At the same time, these same participants had a much more active attitude toward pregnancy and breastfeeding. On the other hand, women who were born and lived in Melilla attended the maternal education program less and relied more on common sense or the help they received from their relatives.

This is my first child, and everything has been very easy, really. A very good pregnancy, a wonderful delivery, and breastfeeding without problems. The truth is, I didn't attend any classes or train in anything. I have 5 sisters, all with children, so I am very experienced with children, and my mother, sisters, and sisters‐in‐law also help me a lot and advise me or solve doubts if I have any.(18L)

#### 3.1.3 Employment

In both groups of participants, returning to work only 4 months after giving birth, or even earlier, made them rethink the type of breastfeeding they practiced. For those who chose artificial or mixed breastfeeding from the beginning, it was not as complicated. But for those who opted for exclusive breastfeeding, it was a great challenge to continue, being away from their children for so many hours. They had to pump milk during work hours, create their own milk banks at home, deal with engorged and painful breasts, etc. For women who work as self‐employed, this type of work is a sufficient reason not to practice breastfeeding.

I am now giving artificial milk. I was on mixed breastfeeding for the first three months, but as I knew I would return to work soon and didn't want my baby to suffer or myself either, I recently started weaning, and we are on artificial now. (30H)

All of them highlighted that returning to work was a great obstacle for breastfeeding and parenting in general. All of them, whether employed or unemployed, identified Spain's maternity leave policies as a great injustice to motherhood.

Returning to work is driving me crazy. Maternity leave should be extended because it doesn't make sense that organizations recommend six months of exclusive and on‐demand breastfeeding, but our laws don't allow us to do so. (19M)

#### 3.1.4 Number of Children

The number of children can influence the chosen feeding method after each childbirth. Not only can previous experience affect the type of breastfeeding, but also the time a woman must spend with each child and, therefore, the type of breastfeeding she will practice.

As I had more children, I breastfed the younger ones for less time. It's true that preparing bottles is more time‐consuming, but the feeding sessions are much shorter and allowed me to get things done at home. (8L)

Muslim women practiced longer breastfeeding periods. Nevertheless, in both groups of participants, as the number of children increased, the duration of exclusive breastfeeding decreased. However, there were few cases where this was not the case, due to a strong desire of the woman or because personal circumstances allowed it.

With my eldest daughter, everything was different. I chose mixed breastfeeding for two months and then continued only with formula. The bottle gave me more independence. But now I only think about breastfeeding my daughter a lot and being able to continue as I am. (7M)

### 3.2 Theme 2: Religious Beliefs and Cultural Influence on the Type of Breastfeeding

Culture not only influences a person's daily life but also the different dimensions of motherhood. Some of the women from both religious beliefs perceived that these beliefs influence their motherhood. On the other hand, for the rest, these same aspects were influenced by different aspects of their culture or existing traditions, not by their religious beliefs. But, ultimately, all of them perceived their decisions about motherhood and breastfeeding as conditioned. And this conditioning did not have a direct and exclusive relationship with the fact that the woman identified as a practicing believer.

I chose breastfeeding because of everything I learned in the classes during my pregnancy, but religion was not a determining factor for me. I consider myself a practicing Christian, but my decision was not influenced by my Christianity at all. (11A)

The Muslim participants who identified as practicing highlighted the instructions given in the Quran about breastfeeding and its recommendation to maintain it for 2 years. In their statements, the influence of this sacred text on their decisions about how to feed their offspring could be recognized.

Of course, my decision to breastfeed, like everything I do in my life, is influenced by my religion. In my religion, it is recommended to breastfeed for up to two years, and although I don't know if I'll reach that point, I am very clear that I have the desire and intention to do so. (12D)

### 3.3 Theme 3: Type of Delivery and Type of Breastfeeding

Women who had a cesarean section needed more help, had less autonomy, and saw their feeding options for their infants affected. Those who delivered vaginally were more independent and experienced fewer physical discomforts, allowing them to manage the postpartum period better emotionally. The limitations caused by cesarean sections complicate the initiation of breastfeeding. There were no differences between Muslim and Christian women in this regard.

My delivery was very tough; it was very long, and the epidural didn't work, so I suffered a lot. I ended up having a cesarean section and couldn't see my daughter until 4 hours later, and at that moment, I was not in a state to breastfeed her. (27C)

### 3.4 Theme 4: Knowledge About Breastfeeding and Support Received

#### 3.4.1 Knowledge About Breastfeeding

Christian women, who attended antenatal classes more frequently, played a more active role in decision‐making about their pregnancy, delivery, and the type of breastfeeding they wanted to practice. In contrast, Muslim women attended antenatal classes less often. The former group perceived that information about breastfeeding increased their confidence when starting to breastfeed, empowered them, and boosted their self‐confidence to do so.

During my pregnancy, my partner and I attended antenatal classes and learned a lot about motherhood and breastfeeding. We believe it is necessary to train before becoming parents because it helps a lot afterward. (17M)

Knowing the benefits of breastfeeding over artificial feeding is a reason perceived as very important, prompting women to start exclusive breastfeeding or at least try, even if they cannot always maintain it.

I am exclusively breastfeeding. I was very clear about it once I knew how good it is. Physiologically, it is the food intended for the baby, the most complete, and I knew it would protect my baby the best. (6Y)

#### 3.4.2 Support at the Beginning and for the Maintenance of Breastfeeding

Participants of both religious beliefs perceived the postpartum period as a time of great vulnerability. For them, during this process, it is not only necessary to have family and partner support at home but also to have access to updated, competent healthcare professionals who can provide culturally adapted care.

While we were in the hospital, both the pediatricians and midwives guided and advised us. At the health center, the midwives showed interest in my health, my concerns about breastfeeding, and the baby's condition during pregnancy and postpartum. (31C)

In any case, the participants noted that having a lactation consultant would be important and helpful as a reference professional to solve breastfeeding‐related problems. Often, they were unclear about whom to approach with these issues, whether the midwife, pediatrician, or nurse. Even so, Christian participants reported seeking more advice from healthcare professionals to address breastfeeding issues or simply to seek support during this process.

I miss having a lactation consultant, not because the team wasn't up to par, but because there should be more dedicated postpartum and breastfeeding consultations with more time. (32M)

Regarding family support, participants from both religious beliefs highlighted two particularly important figures: their partner and their mother. In families where there was a “bottle tradition,” breastfeeding was less supported and even discouraged at the first sign of difficulty.

In my house, we were all given bottles. That's been the tradition forever. When I got home with my daughter and was breastfeeding her, my mother always said that the baby was still hungry and needed a bottle. The bottle culture I've always seen at home has greatly influenced me. (14K)

Partner support is perceived as essential, especially in the first days after birth and in adapting to the new role of mother. For the participants, regardless of religious beliefs, having that support helped maintain breastfeeding during the first months of the baby's life, and even after returning to work.

My husband always helps and supports me with breastfeeding, as he also believes it is the best for our children. At home, we are a team. (21A)

The support from the rest of the family was also highlighted as important by all participants, especially from other women in the family, with the mother being the most significant figure. They perceived that their mother was an essential and unconditional pillar both for starting breastfeeding and for deciding to switch to artificial feeding. Mothers always helped them in their decisions.

It's true that he supports me with breastfeeding and loves to see me nurse, but the one who is always there is my mother. Her support is unconditional and necessary for me and for raising my children. (15L)

In addition to the mother, for Muslim women, the mother‐in‐law is also an important figure within the family. They felt they received a lot of support and help from her after the birth of the child. They also considered her recommendations about the benefits of breastfeeding and how to sustain it for as long as possible.

In fact, I believe that if my mother and my mother‐in‐law, who also supported breastfeeding, hadn't insisted so much, I might have opted for formula from the first month. (12D)

## 4 Discussion

This study aimed to understand the reasons influencing the choice and abandonment of exclusive breastfeeding among women of Christian and Muslim religious beliefs. A variety of reasons emerged, ranging from the women's sociodemographic conditions and the support they received to cultural reasons and those related to childbirth.

The age at which women in this study had their first child indicated that younger women tended to prefer mixed or artificial feeding. This was similarly observed in studies conducted in Bangladesh (Ayesha et al. 2021) and Iran (Dalili et al. 2020), where younger mothers had shorter durations of exclusive breastfeeding. For teenage mothers, the lack of freedom exclusive breastfeeding imposes, the loss of their previous social life, the stress of not being able to do it (Suglo et al. 2024), or the stigma of perceived immaturity and unpreparedness (Severinsen et al. 2024) are main reasons they do not start or soon abandon exclusive breastfeeding. However, other studies show that older mothers also initiate or abandon exclusive breastfeeding less frequently (Islam et al. 2024). Therefore, education and support for achieving successful exclusive breastfeeding should be provided to all women, regardless of age.

The place of origin of the women who participated in this study also mattered. Participants highlighted the influence of being a native of a small city like Melilla compared to another city. This affected their attitude toward exclusive breastfeeding due to sociocultural influences. Due to its geographic peculiarity, Melilla can be understood as a more rural environment, where feeding practices are based on social norms and cultural beliefs typical of rural areas (Uusimäki et al. 2023). This influence may include practices that hinder exclusive breastfeeding, such as prelacteal feeding (Mohamed et al. 2020), or misconceptions about the suitability of feeding newborns only breast milk due to ignorance about its composition and benefits for both mother and child (Nsiah‐Asamoah et al. 2020). Other reasons, such as the lack of maternal support from healthcare personnel and difficulties accessing healthcare services in rural areas, lead to lower initiation or early abandonment of exclusive breastfeeding (Wu et al. 2024; Wood et al. 2023). However, despite the lack of resources and existing barriers, women in rural areas sometimes engage in more exclusive breastfeeding and intend to continue it longer than urban mothers due to their attitude toward breastfeeding and previous experiences (Paramashanti et al. 2023; Mohammed et al. 2023).

Returning to work is a time when women consider the continuity of exclusive breastfeeding. This will happen sooner or later, depending on the length of maternity leave (Gianni et al. 2019). However, only 20% of countries worldwide require companies to offer measures that facilitate exclusive breastfeeding (WHO 2023a, 2023b). Therefore, balancing work schedules with breastfeeding and the availability of a place to express breast milk (Iglesias‐Rosado and Leon‐Larios 2021) may be compounded by comments from partners encouraging the use of formula (Murad et al. 2021). Conversely, in other environments, women who worked outside the home provided longer breastfeeding than those who did not, as a method to ensure better nutrition (Ayesha et al. 2021).

Participants in this study reported that the more children they had, the shorter the duration of breastfeeding, primarily due to the increased workload with each additional child. This coincides with another study conducted in Spain, where having more children was a reason for choosing exclusive breastfeeding but not for maintaining it for a longer time (Martín‐Ramos et al. 2024). However, other studies show no relationship between parity and exclusive breastfeeding (Cramer et al. 2021) or indicate that previous childbirth is a reason for starting exclusive breastfeeding (Chertok et al. 2022). Therefore, the number of previous children is not a reason to assume that a woman will practice exclusive breastfeeding.

In the present study, it was evident that religious beliefs also influence the initiation and maintenance of exclusive breastfeeding. This result is consistent with Rehayem et al. (2020), where Muslim women also perceived it as a religious duty mentioned in the Quran. Not doing so can have spiritual consequences (Mohamed et al. 2020). This difference between Muslim and Christian women aligns with a study in the United States, where Christian women were more likely to exclusively formula‐feed their children at birth and 3 months postpartum (Bernard et al. 2020). The significance of religious beliefs has also been found in other groups, such as Evangelicals or Jehovah's Witnesses (Iglesias‐Rosado and Leon‐Larios 2021). Therefore, religious beliefs can also be a reason for starting exclusive breastfeeding, using religious leaders to achieve it (Gyamfi et al. 2024).

The type of delivery, vaginal or cesarean, was also an influential factor. Women who had vaginal deliveries had fewer limitations to initiate breastfeeding earlier, compared to those who had a cesarean section. The latter can lead to the first feeding provided to the newborn being formula or glucose solution, which complicates the subsequent initiation of exclusive breastfeeding and bonding (Mohamed et al. 2020) and may result in stopping it before the recommended duration (Li et al. 2021). This aligns with the finding that cesarean delivery is an independent risk factor for not initiating exclusive breastfeeding postpartum (Jiravisitkul et al. 2022).

The knowledge women have about exclusive breastfeeding was a reason for starting and maintaining it. Improved knowledge about exclusive breastfeeding from antenatal classes was also found in the study by Iglesias‐Rosado and Leon‐Larios (2021), regardless of the women's religious beliefs (Schwarz et al. 2024). Even improved knowledge about breastfeeding in multiparous women who have previously practiced exclusive breastfeeding can help overcome barriers to its initiation and maintenance (Al‐Thubaity et al. 2023; Chen et al. 2024). However, the study by Ayesha et al. (2021) indicated that those who attended antenatal classes had shorter breastfeeding durations compared to those who did not attend.

Feeling supported, both for initiating and maintaining exclusive breastfeeding, is of great importance. As in the present study, the support from the woman's mother is crucial, especially for continuing this type of feeding (Murad et al. 2021; Dalili et al. 2020; Ken et al. 2023). For Muslim women, exclusive breastfeeding is expected of them, and their family also expects it (Rehayem et al. 2020).

Along with the mother, the woman's partner is also an important figure who can contribute to the initiation of exclusive breastfeeding (Iglesias‐Rosado and Leon‐Larios 2021), may enhance their support to mothers during lactation (Akça et al. 2025), achieve successful exclusive breastfeeding (Wagner et al. 2020), and maintain it for at least the first 4 months of the child's life (Panahi et al. 2022). However, there is a dichotomy among fathers between those who perceive breastfeeding as a shared responsibility and those who see it as solely the woman's responsibility. This is due to religious and traditional gender beliefs, where men are expected to work and provide economic sustenance for the household, while women stay home to care for the children and manage their feeding (Chang et al. 2021).

In this study, Muslim women highlighted the importance of the help received from their mother‐in‐law, which assisted in initiating and maintaining exclusive breastfeeding. This finding aligns with studies conducted in India (Debnath et al. 2021), Pakistan (Riaz et al. 2022), and Bangladesh (Lassi et al. 2019). Even among American women, regardless of religious beliefs, there was a greater risk of not breastfeeding or abandoning breastfeeding if they believed their mother‐in‐law's opinion was of little or no importance, compared to women who stated that their mother‐in‐law's opinion was very important (Wallenborn et al. 2019). Therefore, the influence of the mother‐in‐law has shown to be important both for achieving exclusive breastfeeding and not doing so (Suglo et al. 2024; Moreno‐Ávila et al. 2023).

The support received from healthcare personnel is also crucial, both positively and negatively. This type of support can lead to abandoning breastfeeding if mothers are encouraged to use formula at the slightest problem, especially for first‐time mothers (Rehayem et al. 2020). Even without the appearance of problems, healthcare personnel themselves can encourage the abandonment of exclusive breastfeeding (Iglesias‐Rosado and Leon‐Larios 2021). On the other hand, the support of healthcare personnel can generate both the initiation and maintenance of this type of breastfeeding if the midwife is perceived as the reference professional to receive the needed help (Iglesias‐Rosado and Leon‐Larios 2021). Moreover, they are a fundamental piece for making exclusive breastfeeding a positive experience in the case of premature babies or those with low birth weight (Flacking et al. 2021).

### 4.1 Limitations and Strengths of the Work

This study was conducted with Muslim and Christian women in a specific city where the prevalence of both is almost similar. The results might differ if the study were conducted with women of other religious beliefs or in another city with a greater disparity in believer rates. The research team has no suspicions that the participants' contributions do not reflect their reality, as the field notes collected and incorporated into the data analysis showed consistency between what was said and how it was said. These testimonies could be complemented by the contributions of the women's partners who may be involved to varying degrees in the type of feeding their babies receive.

The research team intends to continue and complete this line of research by incorporating other religious beliefs and developing cultural competence in topics related to maternal and child health, due to the lack of studies conducted on this topic in public health systems.

### 4.2 Recommendations for Further Research

Future research should explore the role and perspectives of other stakeholders, such as partners of breastfeeding women and other close family members, such as mothers. Intervention studies also need to be designed and implemented to improve the cultural competence of health workers caring for women during pregnancy, childbirth, and the postpartum period. It is the nursing and midwifery staff who are most directly involved.

## 5 Conclusions

For both Muslim and Christian women, choosing exclusive breastfeeding is influenced by having their first child at an early age, the woman's place of origin, and having family and healthcare support. Furthermore, for Christian women, attending maternal education during pregnancy is influenced by having practiced this type of breastfeeding with their previous children and being practicing mothers. However, what influences the abandonment of exclusive breastfeeding in both groups of women is returning to work, having a cesarean delivery, and, for Muslim women, not attending maternal education.

Despite the strong scientific evidence on the benefits of exclusive breastfeeding, there are still various barriers that prevent this type of feeding from reaching the recommended rate. The differences in motivations that lead women to initiate and/or abandon exclusive breastfeeding due to religious beliefs or other cultural elements mean that healthcare personnel must adapt their interventions to the groups of women and their partners to whom they are directed. Additionally, factors that influence outcomes can vary depending on the study conducted. Therefore, all women should receive help and support for both the initiation and maintenance of exclusive breastfeeding, regardless of their circumstances.

Nursing and midwifery staff closely monitor pregnancy, childbirth, and the postpartum period. Therefore, they must have the necessary training in exclusive breastfeeding, work on their cultural competence, and play a key role in promoting and supporting the best type of feeding for infants. As societies become increasingly multicultural, nursing and midwifery personnel must adapt to this reality and offer culturally adapted care in maternal and child health.

## 6 Relevance for Clinical Practice

Religious beliefs have a physical, psychological, and social influence on people's lives. Health personnel must develop and improve their transcultural competence, especially in maternity units and primary health care. Due to the lack of knowledge regarding the relationship and influence between religious beliefs and exclusive breastfeeding, women could feel judged during this period.

This article provides information regarding the perceptions of women with Muslim and Christian worldviews before, during, and after exclusive breastfeeding. Religious beliefs are factors that influence when a woman becomes pregnant and modify her perception of breastfeeding. The findings of this study may help improve the care provided to women and families in some aspects of exclusive breastfeeding.

## Author Contributions

Study conception and design: I.M.M.‐Á., J.M.M.‐L., S.G.‐P., S.M.R.‐O., J.O.‐P., S.E.M.‐O., J.C.‐M. Data collection: I.M.M.‐Á. Data analysis and interpretation: I.M.M.‐Á., J.M.M.‐L. Drafting of the article: I.M.M.‐Á., J.M.M.‐L., S.G.‐P., S.M.R.‐O., J.O.‐P., S.E.M.‐O., J.C.‐M. Critical revision of the article: I.M.M.‐Á., J.M.M.‐L., S.G.‐P., S.M.R.‐O., J.O.‐P., S.E.M.‐O., J.C.‐M.

## Ethics Statement

The authors have nothing to report. Nevertheless, authorization was obtained from the coordinator of the Women's Care Unit of the Zona Centro Health Centre (Melilla Health Area, Spain) before data collection began (November 4, 2019). The Melilla Health Area does not have a research ethics committee.

## Consent

All participants had to sign an informed consent form.

## Conflicts of Interest

The authors declare no conflicts of interest.

## Data Availability

The data that support the findings of this study are available on request from the corresponding author. The data are not publicly available due to privacy or ethical restrictions.
